# Drug-Resistant Infections in Burn Patients: A One-Year Analysis of Microbiological Trends and Predictive Risk Factors in a Romanian Tertiary Care Centre

**DOI:** 10.3390/antibiotics15030307

**Published:** 2026-03-18

**Authors:** Oana Izmendi, Baditoiu Luminita, Corina Musuroi, Silvana Vulpie, Delia Muntean, Adela Voinescu, Silvia Ioana Musuroi, Zorin Petrisor Crainiceanu, Panche Taskov, Romanita Jumanca, Monica Licker

**Affiliations:** 1 Doctoral School, “Victor Babes” University of Medicine and Pharmacy, 300041 Timisoara, Romania; 2 Microbiology Department, “Victor Babes” University of Medicine and Pharmacy, 300041 Timisoara, Romania; 3 Multidisciplinary Research Centre of Antimicrobial Resistance, “Victor Babes” University of Medicine and Pharmacy, 300041 Timisoara, Romania; 4 Epidemiology Department, “Victor Babes” University of Medicine and Pharmacy, 300041 Timisoara, Romania; 5 Microbiology Laboratory, “Pius Branzeu” County Clinical Emergency Hospital, 300723 Timisoara, Romania; 6 Discipline of Clinical Skills, Department I Nursing, “Victor Babes” University of Medicine and Pharmacy, 300041 Timisoara, Romania; 7 Burn Unit, Plastic and Reconstructive Surgery Department, Casa Austria, “Pius Brînzeu” Emergency County Clinical Hospital, 300723 Timisoara, Romania; 8 Plastic Surgery Department, “Victor Babes” University of Medicine and Pharmacy, 300041 Timisoara, Romania; 9 Romanian & Foreign Languages Department, “Victor Babes” University of Medicine and Pharmacy, 300041 Timisoara, Romania

**Keywords:** burn unit infections, antimicrobial resistance, extensively drug-resistant, cumulative antibiogram, *Acinetobacter baumannii*, multidrug-resistant organisms, Romania

## Abstract

Background and Objectives: The susceptibility of burn patients to infections with multidrug-resistant organisms (MDROs) is high. The aim of this study is to describe the local patterns of antimicrobial resistance in a Romanian burn unit and to identify risk factors associated with the acquisition of extensively drug-resistant (XDR) pathogens. Materials and Methods: We conducted a one-year, observational, retrospective single-centre cohort study including all burn patients with at least one positive culture admitted to our unit during 2024. In order to identify the pathogens and perform antibiograms, we used routine microbiological diagnostic tests. A multivariable logistic regression model was used to identify XDR risk factors. We also compiled a cumulative antibiogram using the first non-duplicate isolate per patient, following the CLSI M39 guidelines. Results: Among the 180 total admissions, 128 (71.1%) had at least one positive microbiological culture, resulting in 643 bacterial isolates out of 559 samples. The most frequently identified species were *A*. *baumannii*, *P*. *aeruginosa*, *S*. *aureus*, and *K*. *pneumoniae*. We isolated MDROs in 59.37% of patients, and 26.56% had at least one XDR pathogen isolated during hospitalisation. We identified three independent predictors for the isolation of XDR pathogens: a higher Abbreviated Burn Severity Index (ABSI) score (aOR 6.12; *p* = 0.001), hospital length of stay (LOS) (aOR 1.02; *p* = 0.030), and the number of bacterial species identified per sample, representing polymicrobial growth (aOR 5.91; *p* = 0.001). Conclusions: Our findings highlight a significant percentage of MDR and XDR pathogens and provide the foundation for antimicrobial stewardship measures, using the local cumulative antibiogram for empirical therapy.

## 1. Introduction

Burn injuries carry high morbidity and mortality worldwide, and survivors frequently experience long-term sequelae that affect their quality of life and social reintegration. They may be caused by thermal, electrical, or chemical injuries and are often associated with extensive damage to the skin and underlying tissues [1]. The clinical management of these wounds is challenging.

Infections are among the most common complications in burn patients and frequently involve multidrug-resistant organisms (MDROs), which contribute to mortality, particularly in extensive burns and prolonged hospitalisation [2]. Factors that can cause the high occurrence of infections in burn wounds include patient characteristics (age, sex, comorbidities), extent and depth of the burn, inhalation injury, extended admission, repeated use of invasive procedures, exposure to broad-spectrum antibiotics, disruption of the skin barrier, and the immune dysregulation that is usually associated with burn injuries, adding to the selection and transmission of MDROs in burn patients [3,4,5,6,7].

A characteristic temporal pattern of bacterial colonisation and infection can be observed in burn wounds. Gram-positive cocci (GPCs) predominate and are typically isolated in the first days of admission, originating from the patient’s endogenous skin flora, and then progressing to Gram-negative bacilli (GNBs) such as *P*. *aeruginosa*, *A*. *baumannii* and *K*. *pneumoniae*. The latter tend to be isolated after approximately five days of hospitalisation, often originating through acquisition of environmental and healthcare-associated organisms. Skin and soft tissue infections appear during the first week of admission, while pulmonary, urinary, and systemic infections tend to develop later [3,8]. Understanding these dynamics, along with local antimicrobial resistance (AMR) trends, can help optimise empirical therapy and prevent the emergence of MDROs.

In Romania, the epidemiological monitoring and surveillance of MDROs is still inconsistent across the country. At the Pius Brînzeu County Emergency Clinical Hospital (the largest tertiary care centre in western Romania), routine monitoring of MDROs can be challenging due to the low availability of electronic surveillance tools used in tracking AMR and performing cumulative antibiograms. These practical constraints make it more difficult for clinicians to recognise emerging resistance, to detect outbreaks, and to use local evidence to guide empirical antibiotic treatment. This reinforces the need for antimicrobial stewardship (AMS) measures based on the local data, specific to the local epidemiology.

This study is based on a one-year analysis of the bacterial isolates from patients admitted to the Burns Functional Unit (BFU) and has the following objectives: (1) to describe the local microbiological profile and AMR patterns; (2) to examine clinical and demographic risk factors associated with MDRO infections; (3) to identify independent predictors of extensively drug-resistant (XDR) infections through multivariable analysis—to our knowledge, the first such analysis conducted in a Romanian burn unit. The findings will support the optimisation of local antimicrobial guidelines as well as infection prevention and control strategies, and provide a foundation for the development of an AMS initiative within the BFU. We would also like to emphasise the potential role of complementary therapeutic approaches, such as bacteriophage therapy, as future adjuncts in the management of MDRO infections, a subject that will be addressed in subsequent studies.

## 2. Results

### 2.1. Patient Demographics and Clinical Characteristics

A total of 128 burn patients with at least one positive microbiological culture during hospitalisation met the inclusion criteria of our study and were included in the analysis. The cohort was predominantly male (93 men, 72.7%; 35 women, 27.3%), with ages ranging from 18 to 90 years, with a median of 56 [IQR 41–70].

Thermal burns were the leading type of burn, accounting for 118 cases (92.2%). Other less common etiologies included electrical burns, seen in five patients (3.9%); chemical burns, seen in three (2.3%); and mixed injuries, seen in two (1.6%).

The median hospital length of stay (LOS) was 21.5 days [IQR 11–40.75], with a cumulative total of 3880 inpatient days across the entire cohort. When stratified by duration of hospitalisation, 79 patients (61.7%) were hospitalised for 1 to 30 days, 37 (28.9%) for 31 to 60 days, 6 (4.7%) for 61 to 90 days, and an equal proportion (4.7%; *n* = 6) for more than 90 days.

The majority of patients (77.3%, *n* = 99) had deep partial-thickness (II B) to full-thickness (III) burns, while the remaining 29 patients (22.7%) presented superficial to partial-thickness burns (grade I–IIA).

Outcomes at discharge varied, a quarter of patients (34, 26.6%) left the hospital fully recovered, while over half (66, 51.6%) had improved enough to continue treatment as outpatients. Twenty-eight patients (21.9%) died during hospitalisation.

The median Abbreviated Burn Severity Index (ABSI) score was 8 [IQR 6–10], corresponding to the “serious” risk category (estimated survival: 50–70%). Pre-existing comorbidities were documented in 64% of patients. The distributions of ABSI scores at admission, associated comorbidities, and surgical intervention (skin graft surgery) are summarised in Table 1.

### 2.2. Microbiological Profile: Overall Distribution of Samples and Isolates

Out of a total of 559 clinical samples, we recovered 643 non-duplicate bacterial isolates. The higher number of isolates compared to samples reflects the presence of polymicrobial growth in a proportion of samples.

The distribution and frequency of bacterial species isolated from different sample types are summarised in Table 2. Because polymicrobial growth occurred in some samples and the number of isolates exceeds the number of clinical samples, Table 2 reports isolates rather than samples. The four most frequently identified pathogens were *A*. *baumannii*, *P*. *aeruginosa*, *S*. *aureus*, and *K*. *pneumoniae*. The category “Others” includes species with an individual prevalence of below 3%.

#### 2.2.1. Wound Secretions

Wound secretions (*n* = 298) accounted for more than half (53.3%) of all analysed samples and led to 360 bacterial isolates from 19 bacterial genera. The predominant pathogens were *A*. *baumannii*, *S*. *aureus*, *P*. *aeruginosa*, and *Enterococcus* spp. The overall profile included non-fermenting GNBs (30.0%), GPCs (30.8%), and *Enterobacterales* (29%), a relatively balanced distribution across these major bacterial groups (Table 2).

Most wound samples (*n* = 241; 80.9%) were monomicrobial, yielding a single bacterial species, while 52 samples (17.4%) contained two species, and five samples (1.7%) contained three species. Among polymicrobial wounds, the organisms most frequently identified were *A*. *baumannii* (*n* = 23), *S*. *aureus* (*n* = 19), *P*. *aeruginosa* (*n* = 12), *K*. *pneumoniae* (*n* = 9), *Enterococcus* spp. (*n* = 9), and *Enterobacter* spp. (*n* = 9). Rare isolates included one case of *Leclercia adecarboxylata* and one of *Aspergillus fumigatus*.

*Bacillus cereus* was the most frequently isolated organism within the “Others” category (*n* = 35). It is a well-known environmental organism that can contaminate wounds through contact with contaminated objects, equipment, or surroundings in burn units [9], reported to participate in biofilm formation on wound surfaces and medical devices, potentially contributing to more persistent infections.

#### 2.2.2. Bronchial Aspirates

Bronchial aspirates were obtained from 34 intubated, mechanically ventilated patients, one of whom had inhalation injury. All had deep partial- to full-thickness burns (grade IIB–III), with a median ABSI score of 10 [IQR 9–11] and a median LOS of 44.5 days [IQR 28–69], indicating a more severely affected subgroup than the overall cohort.

Bronchial aspirates represented 12.5% of all samples (*n* = 70) and yielded isolates from 14 bacterial genera; polymicrobial growth resulted in a total of 87 isolates from these samples (Table 2). The most prevalent species were *A*. *baumannii*, *P*. *aeruginosa*, *S*. *aureus*, and *K*. *pneumoniae*. Non-fermenting GNBs accounted for approximately 60% of respiratory isolates, while *Enterobacterales* and GPCs represented approximately 20% each.

The majority of bronchial samples (*n* = 53; 75.7%) were monomicrobial, while 17 samples (24.3%) contained two bacterial species. The most frequent bacterial associations were *A*. *baumannii*—*P*. *aeruginosa* (*n* = 4), *A*. *baumannii*—*K*. *pneumoniae* (*n* = 3), and *P*. *aeruginosa*—*S*. *aureus* (*n* = 2). *P*. *aeruginosa* was the most frequently identified organism in polymicrobial respiratory samples (*n* = 8), followed by *A*. *baumannii* (*n* = 7), *S*. *aureus* (*n* = 6), and *K*. *pneumoniae* (*n* = 5).

#### 2.2.3. Blood Cultures

A total of 138 blood cultures (24.7% of all samples) were examined, yielding 142 isolates from 16 bacterial genera. The main species identified were *Staphylococcus* spp. (including both *S*. *aureus* and CNSs), *A*. *baumannii*, and *K*. *pneumoniae*.

Most blood cultures were monomicrobial, with only four samples (2.9%) showing mixed growth of two bacterial species. The bacterial combinations identified were *A*. *baumannii*—*P*. *aeruginosa*, *K*. *pneumoniae*—*S*. *aureus*, and *P*. *aeruginosa*—*Enterococcus* spp.

Fungal bloodstream isolates were rare. However, one case of *Candidozyma auris* bloodstream infection was identified in a 71-year-old woman presenting with flame burns of grade IIB–III and an ABSI score of 10. The isolate was recovered during a 71-day hospitalisation period. The patient was discharged with a good outcome. Infection control measures were implemented following identification, with no local outbreak detected. Additionally, one anaerobic GNB, *Prevotella timonensis*, was also isolated.

#### 2.2.4. Urine Cultures

Urine cultures accounted for 9.3% of all analysed samples (*n* = 52). The most common uropathogen was *A*. *baumannii*, identified in approximately 25% of urine isolates. Fungal infections (predominantly *Candida* spp.) and *E*. *coli* isolates were also numerically significant. This distribution was consistent with the microbiological patterns observed in catheter-associated urinary tract infections among critically ill burn patients, in which environmental and healthcare-associated pathogens often predominate over typical community-acquired uropathogens.

### 2.3. Antimicrobial Resistance Patterns

A cumulative antibiogram of the primary bacterial species isolated from burn patients was generated to guide empirical therapy selection, following CLSI M39 guidelines [10]. Percentages of susceptible isolates were calculated based on the first non-duplicate isolates per patient and bacterial species. The results are presented in Table 3; colour coding is used to facilitate visual interpretation.

Concurrently with the cumulative antibiogram, a resistance phenotype analysis was performed to characterise the epidemiological burden of resistance in the unit, following a modified WHO GLASS methodology [11]. This analysis evaluated the distribution of MDR, XDR, extended-spectrum beta-lactamase (ESBL)-producing, and carbapenem-resistant organisms (CROs). This analysis differs methodologically from the cumulative antibiogram because it includes all non-duplicate isolates (not only the first isolate per patient), allowing a more complete assessment of resistance over the hospitalisation period. The results are summarised in Table 4.

High proportions of MDR and XDR isolates were observed, particularly among *A*. *baumannii* and *K*. *pneumoniae*, with substantial carbapenem resistance. *A*. *baumannii* accounted for the highest absolute number and proportion of XDR strains, while *K*. *pneumoniae* also exhibited a notably high frequency of XDR phenotypes. *P*. *aeruginosa* contributed substantially to the overall MDR burden but showed relatively fewer XDR isolates compared to *A*. *baumannii* and *K*. *pneumoniae*. Among GPCs, methicillin-resistant *S. aureus* (MRSA) and macrolide–lincosamide–streptogramin B (MLSB) resistance phenotypes were frequently observed.

In accordance with the heterogeneous resistance patterns, antibiotic exposure was variable among the 128 patients. Approximately one-third of them (*n* = 40; 31.2%) did not receive systemic antibiotic therapy, while 27 patients (21.1%) received only prophylactic treatment, most commonly cefazolin. Among patients who received therapeutic antibiotics, 14 patients (10.9%) were exposed to two antibiotics (categorised as moderate exposure), and 28 patients (21.9%) received three to five antibiotics (high exposure). A further 19 patients (14.8%) experienced extensive polypharmacy, receiving six or more different systemic agents during hospitalisation. Within this latter group, five patients received six antibiotics, three patients each received seven, eight, nine, or ten, and two patients were treated with as many as 11 antibiotics—the highest number recorded in the cohort. In addition to systemic therapy, 19 patients (14.8%) also received topical antibiotics as part of wound management (topical treatments were not included in the systemic exposure categories). For analytical purposes, all patients who received at least one antibiotic, whether oral, intravenous, or topical, at any point during hospitalisation were classified as having antibiotic exposure.

### 2.4. Distribution of MDR and XDR Isolates by Sample Type

The distribution of MDR and XDR isolates was analysed by bacterial species and sample type to identify potential associations between infection site and AMR level. The frequency of MDR and XDR isolates across different clinical samples is summarised in Table 5.

MDR isolates were identified across all sample categories. Notably, the proportion of MDR isolates was lower in wound secretions compared with bronchial aspirates (*p* = 0.003) and blood cultures (*p* = 0.002), while no statistically significant difference was observed between wounds and urine samples (*p* = 0.435). Similarly, XDR isolates were significantly less frequent in wound samples compared with blood cultures (*p* = 0.011) and urine cultures (*p* = 0.006). The difference between wound and bronchial aspirate samples for XDR pathogens did not reach statistical significance (*p* = 0.066).

### 2.5. Patient-Level Analysis: Distribution of High-Risk Resistance Phenotypes and Risk Factors for XDR Infection

To assess the clinical burden of AMR at the patient level rather than the isolate level, patients were classified according to the highest resistance phenotype identified among all isolates recovered during their hospitalisation (Table 6).

Based on this classification, 59.37% of patients (*n* = 76) harboured MDR strains (including XDR), while 26.56% (*n* = 34) had at least one XDR pathogen isolate identified during hospitalisation. Additionally, 37.5% of patients (*n* = 48) had at least one isolate exhibiting an ESBL or CRO phenotype, and 36.72% (*n* = 47) were identified as having MRSA. These findings underscore the substantial burden of highly resistant pathogens in the burn unit and highlight the limited therapeutic options available for empirical and targeted treatment.

### 2.6. Risk Factor Analysis for XDR Infection

To identify clinical and demographic variables associated with the isolation of XDR pathogens, a univariate analysis was performed comparing XDR patients (*n* = 34) to non-XDR patients (*n* = 94). Several variables were found to be significantly associated with XDR infections in the univariate analysis, including a higher ABSI score at admission (*p* < 0.001), greater burn depth (*p* = 0.001), prolonged length of hospital stay (*p* < 0.001), surgical intervention (skin graft) (*p* < 0.001), the presence of polymicrobial growth (*p* < 0.001), and infections caused by ESBL/CROs (*p* < 0.001) or MRSA (*p* = 0.022). No statistically significant associations were observed for patient sex, age at admission, or pre-existing comorbidities.

Variables meeting the inclusion threshold in the univariate analysis were entered into a multivariate logistic regression model (Table 7) to identify independent predictors of XDR strains after adjusting for potential confounders. In the adjusted analysis, three variables remained independently associated with XDR identification: ABSI score at admission (aOR = 6.12; 95% CI 2.13–17.61; *p* = 0.001), LOS (aOR = 1.02; 95% CI 1.00–1.04; *p* = 0.030), and the number of bacterial species identified per sample, representing polymicrobial growth (aOR = 5.91; 95% CI 2.06–16.95; *p* = 0.001).

The multivariable model demonstrated good fit, with a Hosmer–Lemeshow goodness-of-fit *p*-value of 0.561, and a Nagelkerke R^2^ of 0.469, indicating that the model explained approximately 47% of the variance in XDR infection risk.

## 3. Discussion

In this one-year analysis of 128 patients admitted to the burn unit, we identified 643 bacterial isolates, recovered most frequently from wounds, followed by blood cultures and bronchial aspirates. The most frequent pathogens were *A*. *baumannii*, *P*. *aeruginosa*, *S*. *aureus* and *K*. *pneumoniae*. A high AMR rate was observed, particularly in *A*. *baumannii* and *K*. *pneumoniae*, including resistance to broad-spectrum cephalosporins and carbapenems, as well as methicillin resistance in *S*. *aureus*. These findings confirm prior observations from the same burn unit made during the COVID-19 period [12], and reflect the broader national trends of increased MDRO prevalence and antibiotic consumption reported in Romania during the pandemic [13,14,15,16], which are also consistent with the patterns reported in other burn units, where GNB MDROs predominate [2].

These findings should be considered in the broader context of burn-related morbidity and mortality. Burn injuries continue to be a major public health problem worldwide, responsible for more than 180,000 deaths each year, according to the World Health Organization (WHO) [1]. In addition to high mortality, the burn patients who survive often experience long-term morbidity, including disabling sequelae and disfigurement, with a negative impact on their quality of life [17]. The management of burn wounds is complicated because they are prone to infections caused by MDROs due to their complex pathophysiology.

While these epidemiological patterns are well established in the burn care literature, the present study extends previous reports from this unit [12] by applying multivariate logistic regression to identify independent predictors specifically of XDR, rather than MDR infections. This kind of analysis, to our knowledge, has not been previously conducted in a Romanian burn unit.

In our cohort, the majority of patients were male (72.7%), consistent with the male predominance reported across burn unit populations worldwide. Over half of patients (64%) presented with comorbidities, and thermal burns accounted for over 90% of the cases. Severe injuries were common, with more than three-quarters of patients sustaining second- or third-degree burns, characteristics that are consistent with the injury profiles typically reported in other specialised burn units [18,19,20,21,22].

The cumulative antibiogram we generated in this study provides important data for empirical antibiotic selection and actionable stewardship measures. *A*. *baumannii* and *K*. *pneumoniae* exhibited the lowest overall susceptibility rates to most antimicrobial classes, including carbapenems, extended-spectrum cephalosporins, and β-lactam/β-lactamase inhibitor combinations. The differences observed between the cumulative antibiogram and the resistance phenotype analysis reflect their distinct methodological aims.

The resistance phenotype analysis can offer an overview of the resistance burden during hospitalisation. We identified a significant number of MDROs, in particular, *A. baumannii*, which accounted for the highest proportion of XDR strains, followed by *K*. *pneumoniae*. *P*. *aeruginosa* also contributed considerably to the overall MDR burden. Among GPCs, MRSA and MLSB phenotypes were observed in *S*. *aureus*. *A*. *baumannii* and *K*. *pneumoniae* were the principal contributors to XDR burden, while MDR non-fermenters and GPCs added to the complexity of antimicrobial treatment.

The distribution of XDR based on sample types further supports the clinical relevance of these pathogens. XDR *A*. *baumannii* was isolated predominantly from wound samples and also from blood cultures and bronchial aspirates, consistent with previous reports, which recognised its role in invasive burn infections [23,24,25,26]. XDR *K*. *pneumoniae* isolates were most frequently recovered from blood cultures, followed by wounds and urine. A rise in MDR *Pseudomonas* spp. and *Klebsiella* spp. was also observed in the previous local analysis [12,27], suggesting the persistence of these trends over time.

A considerable proportion of patients were affected by highly resistant pathogens. Approximately one quarter of patients (26.6%) had at least one XDR isolate identified during hospitalisation. The high AMR rate is consistent with the selective antibiotic pressure characteristic of burn units and underscores the limited therapeutic options available.

Three factors were identified as independent predictors of isolations of XDR pathogens in the multivariable analysis, having direct implications for how we stratify risk and target infection prevention in burn care.

The median ABSI in our cohort was 8 [IQR 6–10], corresponding to a survival probability of 50–70%. This could indicate that patients with more severe burns are at the highest risk of acquiring highly resistant infections. The association between ABSI and the isolation of XDR strains can be explained by more extensive burns resulting in greater disruption of the skin barrier, more devitalised tissue susceptible to colonisation, immune dysfunction, and an increased probability of invasive procedures and prolonged antibiotic use. Previous studies have also shown that a higher ABSI can be associated with an increased risk of infection in burn patients [28]. Similarly, studies investigating MDR *A*. *baumannii* and *P*. *aeruginosa* infections have reported that elevated ABSI scores were among the strongest predictors of infection [24,29,30]. Together, these findings support a consistent relationship between burn severity and resistance in burn care, and our results extend this relationship to the XDR phenotype.

Polymicrobial growth emerged as another independent predictor of XDR strain isolation. This variable captures the recovery of multiple bacterial species from the same clinical sample above the positivity threshold, exceeding microbial growth on a three-quadrant plate. Polymicrobial colonisation and infection are known to be a major driver of burn-wound complications [6], with more than half of chronic wounds colonised or infected by multiple bacterial species, forming polymicrobial biofilms that delay healing [31]. *P*. *aeruginosa* and *A*. *baumannii* biofilms have been shown to delay epidermal healing in burn wounds [32]. When *S*. *aureus* and *P*. *aeruginosa* coexist, they can act synergistically by sharing resistance genes, forming biofilms, and amplifying their virulence [33,34]. These interactions support our finding that polymicrobial samples were an independent predictor of XDR infection.

LOS is another independent risk factor associated with the isolation of XDR strains, with a median of 21.5 days (IQR 11–40.75); 61.7% of patients were hospitalised for up to 30 days and 28.9% for 30–60 days, consistent with admission durations reported in comparable burn unit studies [2,35,36]. Prolonged LOS is a known factor associated with increased infection risk in burn patients and has been closely linked to the emergence of resistant GNBs.

The interpretation of LOS should be done with caution, as it can be a predisposing factor for MDRO infection due to prolonged exposure of burn patients to invasive procedures and prolonged use of antibiotics, and at the same time, a consequence of severe infections. Other studies support this bidirectional relationship. Li et al. [36] demonstrated a strong association between prolonged LOS and infections with carbapenem-resistant *K*. *pneumoniae*, and two other studies [35,37] reported a similar pattern for GNBs more broadly. Langeveld et al., in their study [38], also observed that patients with MDRO infections had a significantly longer admission time than those without MDRO infections, highlighting the existing dynamic between LOS and AMR.

The European Centre for Disease Prevention and Control (ECDC), in their most recent report [39], indicates that difficult-to-treat Gram-negative infections drove AMR in the WHO European Region in 2024. Of particular concern is *K*. *pneumoniae*’s rising resistance, with 44% of EARS-Net countries reporting a resistance of ≥50% to third-generation cephalosporins and 24% reporting carbapenem resistance at similar levels, especially in Southern and Eastern Europe. Carbapenem-resistant *A*. *baumannii* is also associated with high mortality, and carbapenem resistance in *P*. *aeruginosa* shows variability, with rates above 50% in 11% of reporting countries.

Another important concern is the “convergence” of hypervirulence and carbapenem resistance in *K*. *pneumoniae*. It has been noticed that in burn units, *K*. *pneumoniae* shows a higher AMR rate than in other critical hospital wards [26], likely reflecting prolonged antibiotic exposure and the vulnerability of burn patients to MDRO infection. Overall, these trends highlight that the available treatment options for MDRO infections are becoming increasingly limited, and there is a pressing need for early recognition of emerging resistance patterns and the development of new treatment options.

Romania reports some of the highest AMR rates in Europe [40], likely influenced by high antibacterial consumption, self-medication, over-the-counter sales, and limited implementation of AMS programmes [41]. In response to this problem, a National Action Plan on Antimicrobial Resistance was launched in 2014, and recently, the National Health Strategy 2023–2030 has been adopted with specific objectives for controlling AMR and healthcare-associated infections [42,43].

Most of the microorganisms identified in this study are listed by the WHO as priority MDR pathogens, requiring urgent measures, research, and novel therapeutic options [44]. One current strategy in the fight against AMR is the implementation of AMS programmes. The role of these programmes is to promote the appropriate use of antibiotics, at the correct dose, at the right time, and for the right patient. AMS initiatives have been recognised as essential components of institutional and global strategies to preserve antibiotic efficacy and improve patient outcomes [45]. Our evaluation of bacterial trends and AMR in the BFU represents a foundational step towards a formal AMS initiative, in alignment with the European Society of Clinical Microbiology and Infectious Diseases (ESCMID) AMS Program [46]. The cumulative antibiogram and AMR data will serve as baseline metrics for a planned AMS project in the BFU.

Antibiotic exposure in our cohort was substantial. Comparable patterns of antibiotic use have been reported in other burn centres. In a study by Wermine et al. [47], 32.4% of patients received antibiotics in the first 7 days after injury. These findings highlight the important role of AMS programmes in supporting rational prophylaxis and appropriate escalation and de-escalation. At our institution, a Medication Committee has recently been established to review antibiotic prescribing practices, and local guidelines for antimicrobial use are currently under development.

For the treatment of burn wounds infected by MDROs, a combination of strategies could be used. These include preventive measures, rapid diagnostics, and systemic antibiotics, together with non-antibiotic methods such as topical antimicrobial agents, probiotics, wound dressings, vacuum-assisted systems, and bacteriophages. A combined approach of conventional therapy and new strategies is likely to improve outcomes in burn patients. Numerous studies [2,48,49] have examined these methods as alternative treatments for resistant burn infections.

Reducing the bacterial load and biofilm formation decreases the risk of infections with MDR pathogens and improves wound healing. Several strategies used to reduce biofilm in burn wounds, including silver sulfadiazine, modern disinfectants, hydrogels, lactobacilli, and bacteriophages, are highlighted by Thomas et al. [50]. Phage therapy has been long established in countries like Georgia, Poland, and Russia [22,51,52,53,54] and in recent years has been explored in Western countries as well, representing a potential alternative approach for MDRO infections. Phages have been used in wound decolonisation, with experimental and clinical studies reporting activity against infected and biofilm-associated wounds [55,56,57,58]. Clinical studies on phage therapy in burn wound infections have shown safety and promising results [59,60,61,62,63,64].

### Limitations, Strengths and Future Perspectives

This study has limitations that need to be acknowledged. It is a one-year, retrospective, single-centre study, and the results may not apply to other burn units or time periods.

The analysis was restricted to culture-positive patients. Antibiotic exposure was described categorically (by number of agents and exposure levels) but was not included in the statistical analysis, and the absence of molecular typing methods limits the evaluation of clonal transmission dynamics. Additionally, the differentiation between colonisation and clinically significant infection was based on semi-quantitative microbiological criteria combined with clinical assessment.

Despite these limitations, the study offers an overview of local AMR and pathogen trends, and represents one of the few systematic analyses of MDR and XDR infections in Romanian burn patients. The findings provide a starting point for AMS initiatives and exploration of alternative therapeutic strategies. The epidemiological surveillance of MDROs is an essential component of AMR prevention and control measures. Future studies will address detailed antibiotic consumption and molecular typing methods to strengthen surveillance strategies and support AMS measures in the BFU.

Also, the findings of the present study provide a framework for addressing antimicrobial resistance and alternative therapeutic approaches for MDRO infections. A collection of bacteriophages with lytic activity against XDR *K. pneumoniae* strains isolated from our hospital has been established, and further work will explore their potential epidemiological and therapeutic applications.

## 4. Materials and Methods

### 4.1. Study Design and Setting

We performed a retrospective, single-centre cohort, observational study at the Pius Brînzeu County Emergency Clinical Hospital, a tertiary care centre located in western Romania with a capacity of 1100 beds. In the analysis, we included all patients admitted to the BFU between 1 January and 31 December 2024 who had at least one bacteria- or fungus-positive culture during their hospitalisation.

The unit has 9 beds in total, among which 5 are intensive care unit (ICU) beds in individual isolation rooms, and 4 are distributed across 2 rooms and reserved for patients with intermediate or minor burns. All patients received burn wound care in accordance with local protocols. Antibiotic prophylaxis and treatment in the BFU are guided by institutional guidelines based on the *Sanford Guide to Antimicrobial Therapy* [65]. For surgical prophylaxis, cefazolin is administered 30 min before the surgical procedures; vancomycin is used as an alternative in patients with β-lactam allergy. In patients with documented infections prior to surgery, prophylaxis is directed towards the identified causative pathogens; when antibiotic therapy is already in progress at the time of intervention, the current regimen is continued. Therapeutic antibiotic management is carried out according to the Sanford guidelines, adapted to individual AST results, and following interdisciplinary consultation with infectious disease and clinical microbiology specialists. As part of routine infection prevention measures, surveillance swab cultures were performed at admission and at regular intervals. These screening cultures were excluded from the study, which was restricted to clinical cultures obtained on the basis of clinical suspicion of infection.

### 4.2. Patient Selection and Clinical Data Collection

During the study period, a total of 180 patients were admitted to the BFU. For 128 (71.1%) patients, at least one microbiological culture was positive during their admission, and they were included in the final analysis. The remaining 52 patients (28.9%) had consistently negative cultures throughout hospitalisation and were included only in the overall cohort description but excluded from the microbiological and AMR analyses.

The criteria for inclusion in the study were: (1) an age of ≥18 years; (2) at least one positive bacterial or fungal isolate recovered from clinical samples obtained during hospitalisation.

We retrospectively extracted all clinical, demographic, microbiological, and treatment data from the hospital’s electronic medical records and microbiology laboratory databases. The collected variables are presented in Table 1.

Burn depth was classified according to the European grading system: Grade I (superficial/epidermal), Grade IIA (superficial partial-thickness), Grade IIB (deep partial-thickness), and Grade III (full-thickness)

Burn severity was assessed using the ABSI score [66], with five prognostic categories: 2–5 (we combined the two lowest-risk categories due to limited patient numbers and similar favourable outcomes), 6–7, 8–9, 10–11, and ≥12.

During the study, a total of 559 clinical samples were collected and analysed, yielding 643 bacterial isolates. To avoid duplication, we included only non-duplicate bacterial isolates in the analysis. Non-duplicate isolates were defined as: (1) the first isolate of each bacterial species per patient; (2) subsequent isolates of the same species from the same patient that exhibited a distinct resistance phenotype, or were obtained from samples collected at intervals of at least 10 days. Repeated isolates of the same species from the same patient and anatomical site without phenotypic variation were considered duplicates and excluded.

We used two distinct criteria for the cumulative antibiogram and resistance phenotype analysis. For the cumulative antibiogram, only the first non-duplicate isolate per patient and species was included, and we followed the CLSI M39 guidelines [10] to guide empirical therapy selection. For the resistance phenotype analysis, we adapted the WHO GLASS methodology [11] by including all non-duplicate isolates across admission to capture the full burden of resistance encountered during hospitalisation, consistent with previously validated surveillance methods [67].

All patients (or their legal representatives, in cases of unconscious or critically ill patients at admission) provided written informed consent for the use of their clinical and paraclinical data for research purposes. All data were anonymised prior to analysis to ensure patient confidentiality.

### 4.3. Microbiological Methods and Antibiotic Susceptibility Testing

All cultures included in this analysis were clinically indicated, collected when infection was suspected based on clinical signs. The decision to obtain cultures was made by the treating physician. Samples were classified by anatomical site and included: respiratory tract, bloodstream, wound, and urinary samples. All analysed samples were collected under standard aseptic conditions and transported promptly to the hospital microbiology laboratory.

The differentiation between colonisation and clinically significant infection was based on semi-quantitative assessment of microbial density in combination with clinical data. For wound swab cultures, a standard four-quadrant streaking technique was used, and cultures were considered positive when bacterial growth was observed in at least three quadrants. Growth restricted to fewer than three quadrants was interpreted as colonisation and excluded from the analysis. These microbiological criteria were complemented by clinical assessment.

The samples were inoculated onto standard culture media, including Blood agar, Mannitol Salt agar, MacConkey agar, Chromogenic agar, and Sabouraud dextrose agar (Thermo Fisher Scientific, Oxoid Deutschland GmbH, Wesel, Germany). All blood cultures were collected in aerobic and anaerobic bottles and incubated for up to five days at 37 °C using a BD BACTEC™ monitoring system (Becton Dickinson, Franklin Lakes, NJ, USA).

Bacterial identification was performed using matrix-assisted laser desorption/ionisation time-of-flight mass spectrometry (MALDI-TOF MS) (Bruker Daltonics, Bremen, Germany) and the VITEK^®^ 2 system (bioMérieux, Marcy-l’Étoile, France), using identification cards for Gram-positive (GP) and Gram-negative (GN) bacteria and yeast (YST). Antimicrobial susceptibility testing (AST) was performed using AST cards specific for GN and GP bacteria and for YST or, where appropriate, by the Kirby–Bauer disc diffusion method. The interpretation of AST results was done according to the EUCAST guidelines (version 14.0, 2024).

### 4.4. Phenotypic Classification of Antimicrobial Resistance

Bacterial isolates were phenotypically classified according to standardised resistance definitions based on the AST results and criteria proposed by Magiorakos et al. [68]. MDR isolates were defined as exhibiting acquired non-susceptibility to at least one agent in three or more antimicrobial categories. XDR isolates were defined as those non-susceptible to at least one agent in all but two or fewer antimicrobial categories (i.e., remaining susceptible to only one or two antimicrobial categories).

Extended-spectrum β-lactamase (ESBL)-producing isolates were identified in GNBs that demonstrated resistance to third- and fourth-generation cephalosporins (e.g., ceftriaxone, cefotaxime, ceftazidime, cefepime) or monobactams (aztreonam), with a positive β-lactamase inhibitor synergy test. Carbapenem-resistant organisms (CROs) were defined by phenotypic resistance to at least one carbapenem (imipenem, meropenem, or ertapenem). Methicillin-resistant *S*. *aureus* (MRSA) was identified by resistance to oxacillin or cefoxitin, according to the EUCAST guidelines [69,70]. The macrolide–lincosamide–streptogramin B (MLSB) resistance phenotype was recorded for staphylococcal isolates exhibiting co-resistance to macrolides, lincosamides and streptogramin B antibiotics.

For patient classification, the following definitions were used:

- MDR patient: a patient for whom all bacterial isolates recovered during hospitalisation exhibited an MDR phenotype.
- XDR patient: a patient for whom the most resistant phenotype identified among all isolates during hospitalisation was XDR.
- ESBL/CRO patient: a patient in whom at least one isolate demonstrated an ESBL-producing or carbapenem-resistant phenotype.
- MRSA patient: a patient with at least one MRSA isolate detected during hospitalisation.

### 4.5. Statistical Analysis

Descriptive statistics were used to summarise patients’ characteristics and microbiological findings. Continuous variables were expressed as the median and interquartile range (IQR) due to a non-normal distribution, while categorical variables were expressed as absolute frequencies and percentages (*n*, %); where appropriate, 95% confidence intervals (95% CI) were calculated using the Wilson method for proportions. The normality of continuous variables was assessed using the Kolmogorov–Smirnov test. Comparison between groups was performed using Student’s *t*-test for normally distributed data or the Mann–Whitney U test for non-normally distributed data. Categorical variables were compared using the Chi-squared test (Fisher’s exact test).

Univariate analysis was performed in order to identify clinical and demographic variables associated with XDR infection. Variables with a *p*-value ≤ 0.05 in univariate analysis were subsequently entered into a multivariable logistic regression model to identify independent predictors of XDR pathogen isolation. Model performance was assessed using the Hosmer–Lemeshow goodness-of-fit test, and the proportion of variance explained was quantified using the Nagelkerke R^2^ coefficient. To avoid multicollinearity among predictor variables, variance inflation factor (VIF) values were calculated using linear regression.

Statistical significance was set at *p* < 0.05 for all tests. All analyses were two-tailed and conducted using IBM SPSS Statistics software version 20 (SPSS Inc., Chicago, IL, USA).

## 5. Conclusions

Burn patients presenting with high ABSI scores, prolonged hospitalisation, and polymicrobial growth represent a subgroup of patients at increased risk for XDR infections. In clinical practice, these patients should be considered high-risk, and empirical antibiotic treatment should be tailored to local resistance patterns and reassessed once microbiological results become available. At the same time, AMS and infection control measures remain essential for the preservation of the limited therapeutic options available.

## Figures and Tables

**Table 1:** ABSI scores at admission, and comorbidities associated with burn injury.

Variable	Frequency (*n*)	Percent (%)	95% CI
ABSI Score at Admission			
Low–moderate (2–5)	27	21.09	14.92–28.95
Moderate–severe (6–7)	33	25.78	18.99–33.99
Serious (8–9)	34	26.56	19.68–34.82
Severe (10–11)	19	14.84	9.71–22.02
Maximum (≥12)	15	11.72	7.23–18.44
Associated Comorbidities			
No comorbidities	47	36.72	28.87–45.34
Metabolic and hepatic diseases	11	8.59	4.87–14.73
Respiratory and cardiovascular diseases	35	27.34	20.37–35.64
Combined metabolic/hepatic and cardiovascular/respiratory disease	20	15.63	10.35–22.90
Others (neurological, psychiatric)	15	11.72	7.23–18.44
Grafting			
No grafting	54	42.19	33.98–50.85
Grafting	74	57.81	49.15–66.02

Confidence intervals were calculated using the Wilson method.

**Table 2:** Distribution of microbial species by sample type [*n* (% within sample type)].

	Wound Isolates (*n* = 360)	Bronchial Aspirates (*n* = 87)	Blood Cultures (*n* = 142)	Other (*n* = 2)	Total (*n* = 643)
*A*. *baumannii*	63 (17.50)	30 (34.48)	27 (19.01)	1 (50.00)	134 (20.84)
*S*. *aureus*	52 (14.44)	10 (11.49)	12 (8.45)	-	74 (11.51)
*P*. *aeruginosa*	43 (11.94)	20 (22.99)	13 (9.15)	-	83 (12.91)
*Enterococcus* spp.	38 (10.56)	-	13 (9.15)	-	54 (8.40)
*K*. *pneumoniae*	29 (8.06)	7 (8.05)	21 (14.79)	1 (50.00)	66 (10.26)
*E*. *coli*	26 (7.22)	4 (4.60)	1 (0.70)	-	40 (6.22)
*Enterobacter* spp.	23 (6.39)	-	6 (4.23)	-	29 (4.51)
*Proteus* spp.	14(3.88)	1 (1.15)	1 (0.70)	-	17 (2.64)
CNSs	8 (2.22)	-	22 (15.49)	-	30 (4.67)
*Corynebacterium* spp.	6 (1.67)	6 (6.90)	7 (4.93)	-	19 (2.95)
Fungi	3 (0.83)	3 (3.45)	9 (6.34)	-	26 (4.04)
Others	69 (19.17)	7 (8.05)	11 (7.75)	-	88 (13.69)

Abbreviations: CNSs = coagulase-negative staphylococci. “Others” includes: *Bacillus cereus* (*n* = 35), *Morganella morganii* (*n* = 4), *Providencia stuartii* (*n* = 1), *Pantoea agglomerans* (*n* = 3), *Leclercia adecarboxylata* (*n* = 1), *Prevotella timonensis* (*n* = 1), and *Citrobacter freundii* (*n* = 1). “-” indicates not applicable. The “Others” sample category refers to a single vaginal secretion sample. Percentages were calculated using the total number of isolates recovered from each sample type (wounds *n* = 360, bronchial aspirates *n* = 87, blood cultures *n* = 142, others *n* = 2) as denominators.

**Table 3:** Cumulative antibiogram % sensitivity for GNBs and GPCs.

GNBs (*n*)		AMP	AMC	TCC	TZP	CXM	CTX	CEFT	CAZ	FEP	CZA	CPT	IMI	MEM	AK	GN	TOB	CS	CIP	SXT
*A. baumannii* (31)													0	0	6	6	0	90	0	
*Escherichia coli* (31)			52		90	74		81	81	84			97	97	97	85			82	52
*Enterobacter* spp. (20)				71	93		90		90	90			94	95	100	95	90		85	90
*K. pneumoniae* (23)			43		57		43		43	43	64	59	65	61	61	68	53	78	43	45
*P. aeruginosa* (31)					58				77	77	81	81	70	77	84			90	74	
*Proteus* spp. (13)		27	42		92		85		85	92			77	100	100	77			75	20
**GPB** (***n***)		**P**	**AMP**	**OXA**		**VA**	**TEC**	**GN**	**GnH**	**TE**	**Tige**	**LZD**	**Q/D**	**ER**	**DA**	**CIP**	**LEV**	**Mfx**	**SXT**	**FA**
*S. aureus* (55)		9		49		100	95	96		62	100	100	100	33	40	90	94	90	96	84
*Enterococcus* spp. (39)			100			100	100		87		95	97				76				
	≥90% sensitivity			70–89% sensitivity							<70% sensitivity					IR = intrinsic resistance				

Blank spaces—not applicable or not tested; AMP—ampicillin; AMC—amoxicillin/clavulanic acid; TCC—ticarcillin/clavulanic acid; TZP—piperacillin/tazobactam; CXM—cefuroxime; CTX—cefotaxime; CEFT—ceftriaxone; CAZ—ceftazidime; FEP—cefepime; CZA—ceftazidime/avibactam; CPT—cefpirome; IMI—imipenem; MEM—meropenem; AK—amikacin; GN—gentamicin; TOB—tobramycin; CS—colistin; CIP—ciprofloxacin; SXT—trimethoprim/sulfamethoxazole; Percentages represent the proportion of susceptible isolates per antibiotic based on the cumulative antibiogram of non-duplicate clinical isolates collected from burn patients during 2024. For bacterial species with fewer than 30 isolates, susceptibility percentages should be interpreted with caution due to the limited sample size.

**Table 4:** Distribution of resistance phenotypes by bacterial species.

Bacteria (*n*)	MDR *n* (%)	XDR	ESBL	CROs	R-AG	R-FQ	R-SULF	MRSA	MLSB
*A*. *baumannii* (134)	129 (96.27)	113 (84.33)	-	127 (94.78)	113 (84.33)	128 (95.52)	127 (94.78)	-	-
*P*. *aeruginosa* (83)	47 (56.63)	5 (6.02)	-	20 (24.10)	6 (7.23)	21 (25.30)	-	-	-
*K*. *pneumoniae* (66)	42 (63.64)	37 (56.06)	41 (62.12)	36 (54.55)	27 (40.91)	40 (60.61)	39 (59.09)	-	-
*S*. *aureus* (74)	43 (58.11)	0 (0.00)	-	-	2 (2.70)	4 (5.41)	2 (2.70)	38 (51.35)	42(56.76)
Total *n* = 643	261 (40.59 *)	155 (24.11 *)	41 (6.38 *)	183 (28.46 *)	148 (23.02 *)	193 (30.02 *)	168 (26.13 *)	38 (5.91 *)	42 (6.53 *)

Values are presented as absolute counts, followed by the percentage within species in parentheses. MDR, multidrug-resistant; XDR, extensively drug-resistant; ESBL, extended-spectrum β-lactamase-producing; CROs, carbapenem-resistant organisms; R-AG, resistance to aminoglycosides; R-FQ, resistance to fluoroquinolones; R-SULF, resistance to sulphonamides; MRSA, methicillin-resistant *S*. *aureus*; MLSB, macrolide–lincosamide–streptogramin B resistance phenotype; “-” indicates not applicable for that species; % represents the percentage within spp. * Total row percentages are calculated against all 643 isolates in the study to show the proportion of the overall resistance burden attributable to these four species.

**Table 5:** MDR/XDR isolate distribution by sample type.

Samples		A.B	K.P	P.A	S.A	CNS	CoR	E.C	EnB	PrT	SeR	St.M	Others	Total
Wounds (W) *n* = 360 (% within W)	MDR	58 (16.11)	16 (4.44)	29 (8.06)	29 (8.06)	5 (1.39)	6 (1.67)	9 (2.50)	3 (0.83)	4 (1.11)	3 (0.83)	-	11 (3.06)	173 (48.06)
	XDR	52 (14.44)	12 (3.33)	3 (0.83)	-	-	-	3 (0.83)	-	1 (0.28)	-	-	-	71 (19.72)
Bronchial aspirate (BA) *n* = 87 (% within BA)	MDR	30 (34.48)	1 (1.15)	9 (10.34)	6 (6.90)	-	6 (6.90)	3 (3.45)	-	-	1 (1.15)	1 (1.15)	-	57 (65.52)
	XDR	23 (26.44)	1 (1.15)	-	-	-	-	-	-	-	-	1 (1.15)	-	25 (28.74)
Blood culture (BC) *n* = 142 (% within BC)	MDR	27 (19.01)	17 (11.97)	4 (2.82)	9 (6.34)	19 (13.38)	6 (4.23)	1 (0.70)	5 (3.52)	1 (0.70)	-	-	1 (0.70)	90 (63.38)
	XDR	25 (17.61)	17 (11.97)	1 (0.70)	-	-	-	-	-	-	-	-	-	43 (30.28)
Urine culture (U) *n* = 52 (% within U)	MDR	13 (25.00)	7 (13.46)	5 (9.62)	-	-	-	3 (5.77)	-	-	-	-	-	28 (53.85)
	XDR	12 (23.08)	6 (11.54)	1 (1.92)	-	-	-	-	-	-	-	-	-	19 (36.54)
Others (O) *n* = 2 (% within O)	MDR	1 (50.00)	1 (50.00)	-	-	-	-	-	-	-	-	-	-	2 (100.00)
	XDR	1 (50.00)	1 (50.00)	-	-	-	-	-	-	-	-	-	-	2 (100.00)
Total strains *n* = 643 (% within Total)	MDR	129 (20.06)	42 (6.53)	47 (7.31)	44 (6.84)	24 (3.73)	18 (2.80)	16 (2.49)	8 (1.24)	5 (0.78)	4 (0.62)	1 (0.16)	12 (1.87)	350 (54.43)
	XDR	113 (17.57)	37 (5.75)	5 (0.78)	-	-	-	3 (0.47)	-	1 (0.16)	-	1 (0.16)	-	160 (24.88)

A.B (*A*. *baumannii*), K.P (*K*. *pneumoniae*), P.A (*P*. *aeruginosa*), S.A (*S*. *aureus*), CNSs (coagulase-negative staphylococci), CoR (C*orynebacterium* spp.), E.C (*Escherichia coli*), EnB (*Enterobacter* spp.), PrT (*Proteus* spp.), SeR (*Serratia* spp.), St.M (*Stenotrophomonas maltophilia*). “-” indicates not applicable. “Others” includes infrequently isolated species such as *Bacillus cereus*, *Morganella morganii*, *Providencia stuartii*, *Pantoea agglomerans*, *Leclercia adecarboxylata*, *Prevotella timonensis,* and *Citrobacter freundii*. For species with <10 isolates, percentages are reported for completeness but should be interpreted cautiously due to small sample size.

**Table 6:** Distribution of patients according to resistance phenotypes.

Resistance Phenotype	Frequency (*n*)	Percentage (%)	95% IC
Non-MDR	52	40.62	32.51–49.29
MDR (including XDR)	76	59.37	50.71–67.49
XDR	34	26.56	19.68–34.82
ESBL/CRO Phenotype			
Without ESBL/CRO phenotype	80	62.50	53.86–70.41
With ESBL/CRO phenotype	48	37.50	29.59–46.14
MRSA Status			
Without MRSA	81	63.28	54.66–71.13
With MRSA	47	36.72	28.87–45.34

**Table 7:** Multivariate analysis of risk factors for isolation of XDR strains.

	Univariate Analysis			Multivariate Logistic Regression	
Parameter	*p*-Value	OR	95% CI	*p*-Value	aOR [95% CI]
Patient sex	0.894	0.942	[0.388–2.283]		
Age at admission	0.342	0.675	[0.300–1.522]		
ABSI score at admission	<0.001	8.163	[3.358–19.843]	0.001	6.12 [2.13–17.61]
Burn depth	0.001	14.000	[1.824–107.449]		
Grafting	<0.001	4.549	[1.977–10.467]		
Associated comorbidities	0.148	1.885	[0.793–4.482]		
Length of hospital stay	<0.001	41.167	[7.373–229.840]	0.030	1.02 [1.00–1.04]
Discharge status (deceased vs. healing/healed)	0.007	3.26	[1.35–7.89]		
ESBL/CRO infections	<0.001	188.57	[23.82–1492.73]		
MRSA infections	0.022	2.522	[1.129–5.630]		
No. of bacterial species identified per sample	<0.001	10.63	[4.22–26.83]	0.001	5.91 [2.06–16.95]

OR—Odds Ratio; CI—confidence interval; aOR—adjusted Odds Ratio; ABSI—Abbreviated Burn Severity Index; MRSA—methicillin-resistant *S*. *aureus*; ESBL—extended-spectrum beta-lactamase; CRO—carbapenem-resistant organism. Variables included in the model are shown regardless of statistical significance to illustrate the complete adjustment strategy.

## Data Availability

The original contributions presented in this study are included in this article. Further inquiries can be directed to the corresponding author.

## Abbreviations

The following abbreviations are used in this manuscript:

ABSI	Abbreviated Burn Severity Index
AMS	Antimicrobial stewardship
BFU	Burns Functional Unit
CRO	Carbapenem-resistant organism
ESBL	Extended-spectrum beta-lactamase
EUCAST	European Committee on Antimicrobial Susceptibility Testing
GNBs	Gram-negative bacilli
GPCs	Gram-positive cocci
MDR	Multidrug-resistant
MDRO	Multidrug-resistant organism
MLSB	Macrolide–lincosamide–streptogramin B
MRSA	Methicillin-resistant *S. aureus*
WHO	World Health Organization
XDRWHO	Extensively drug-resistant World Health Organization
XDR	Extensively drug-resistant
